# Apocynin Dietary Supplementation Delays Mouse Ovarian Ageing

**DOI:** 10.1155/2019/5316984

**Published:** 2019-10-20

**Authors:** F. Timóteo-Ferreira, S. Mendes, N. A. Rocha, L. Matos, A. R. Rodrigues, H. Almeida, E. Silva

**Affiliations:** ^1^Instituto de Biologia Molecular e Celular (IBMC) and Instituto de Inovação e Investigação em Saúde (I3S), Universidade do Porto, Portugal; ^2^Unit of Experimental Biology, Department of Biomedicine, Faculty of Medicine of Porto University, Portugal; ^3^Faculty of Dental Medicine of Porto University, Portugal; ^4^Faculty of Nutrition and Food Sciences of Porto University, Portugal; ^5^Obstetrics-Gynecology, Hospital-CUF Porto, 4100 180 Porto, Portugal

## Abstract

Advanced maternal age is associated with higher infertility rates, pregnancy-associated complications, and progeny health issues. The ovary is considered the main responsible for these consequences due to a continuous decay in follicle number and oocyte quality. Intracellular imbalance between oxidant molecules and antioxidant mechanisms, in favour of the former, results in oxidative stress (OS) that is believed to contribute to ovarian ageing. This work is aimed at evaluating whether an age-related increase in ovarian OS, inflammation, and fibrosis may contribute to tissue dysfunction and whether specific antioxidant supplementation with a NADPH oxidase inhibitor (apocynin) could ameliorate them. Mice aged 8–12 weeks (reproductively young) or 38-42 weeks (reproductively aged) were employed. Aged mice were divided into two groups, with one receiving apocynin (5 mM) in the drinking water, for 7 weeks, upon which animals were sacrificed and their ovaries collected. Ovarian structure was similar at both ages, but the ovaries from reproductively aged mice exhibited lipofuscin deposition, enhanced fibrosis, and a significant age-related reduction in primordial and primary follicle number when compared to younger animals. Protein carbonylation and nitration, and markers of OS were significantly increased with age. Moreover, mRNA levels of inflammation markers, collagens, metalloproteinases (MMPs), and tissue inhibitor MMPs (TIMPs) were upregulated. Expression of the antifibrotic miRNA29c-3p was significantly reduced. Apocynin supplementation ameliorated most of the age-related observed changes, sometimes to values similar to those observed in young females. These findings indicate that there is an age-related increase in OS that plays an important role in enhancing inflammation and collagen deposition, contributing to a decline in female fertility. Apocynin supplementation suggests that the imbalance can be ameliorated and thus delay ovarian ageing harmful effects.

## 1. Introduction

During the last decades, developed and developing countries have experienced economic and educational changes that gave women the opportunity to reach higher professional and decision levels. As a consequence, childbearing has been postponed into a period of life when fertility success decreases and pregnancy-associated disorders increase significantly [1].

Human female fertility peaks in the early 20s and gradually declines until the mid-30s. Thereafter, reproductive potential falls sharply, until it virtually ends at menopause around the age of 50 [2]. The ovary is believed to be the main fertility regulator due to the continuous age-related decay in follicle number and oocyte quality [3].

A theory for ovarian ageing holds that age-related disruption of redox homeostasis affects oocyte quality [4]. The continued generation of reactive oxygen species (ROS) together with an age-related decline in activity and expression of important follicle antioxidant enzymes results in an imbalance between ROS production and antioxidant defences. The condition leads to oxidative stress (OS) responsible for protein, amino acid, lipid, and DNA damage that underlie ovarian ageing and fertility loss [5]. As a matter of fact, in follicular fluid of older women, the expression of important antioxidant enzymes, as catalase and specific glutathione S-transferases, is significantly lower when compared with younger women [6]. Similarly, superoxide dismutase 1, superoxide dismutase 2, and catalase gene expression in granulosa [7] and *cumulus oophorus* cells [8] are also downregulated during reproductive ageing. In this process, the use of specific antioxidant molecules has shown beneficial effects in delaying follicle depletion and fertility impairment [9–12].

Studies on ovarian ageing have expanded from the oocyte immediate surroundings to the ovarian stroma, mainly composed of an arrangement of extracellular matrix components (ECM) and fibroblasts and smooth muscle, endothelial, and immune cells. This microenvironment has an important impact on follicle development and oocyte quality. In fact, Briley et al. [13] verified ovarian microenvironment changes with age, specifically an increase in fibrosis and inflammation, and suggested its contributory role to the coincident decrease in oocyte quality. ROS are also believed to contribute to the synthesis and activation of various cytokines and growth factors, hence creating common feedforward and feedback mechanisms that promote tissue fibrosis [14].

Inflammation and ROS formation appear to be key factors in the pathogenesis of ovarian fibrosis [15], which reflects a disturbance of the synthesis and degradation of extracellular matrix (ECM) favouring excessive collagen deposition. Important regulators of ECM homeostasis are metalloproteinases (MMPs), a group of enzymes capable of degrading all types of ECM components [16], and tissue inhibitor metalloproteinases (TIMPs), both affected by ageing [17]. More recent studies have also identified specific microRNAs (miRNAs) as important mediators in fibrosis; they act either by regulating target genes involved in the process of ECM remodelling or by signalling pathways associated with it [18]. Reduction of ROS production by inhibiting NADPH oxidase (NOX) activity with apocynin has shown beneficial effects on renal [19, 20], cardiac [21], skeletal muscle [22], and pulmonary [23] fibrosis. Despite our previous results showing beneficial effects of apocynin supplementation on uterine ageing and fertility [24], its effect on ovarian redox imbalance, inflammation, and fibrosis during reproductive ageing is scarce.

In sum, an age-related low-grade inflammatory state may associate with ovarian collagen deposition and a progressive reduction in follicle number and oocyte quality. In this setting, it was hypothesized that an age-related increase in ovarian fibrosis and ROS underlies fertility reduction. To verify it, markers of oxidative stress, tissue fibrosis, and inflammation, along with the possibility to ameliorate those features by a specific antioxidant supplementation, were evaluated *in vivo*.

## 2. Material and Methods

### 2.1. Animal Handling and Ovarian Tissue Collection

All the experiments were performed according to the Portuguese law on animal welfare and according to the guidelines issued by Federation of European Laboratory Animal Science Associations (FELASA). Female mice (C57BL/6J strain) obtained from Harlan were kept under controlled conditions (12 h light/dark cycle and room temperature at 22°C) and had free access to tap water and standard mouse chow. Young (8-12 weeks old) and reproductively aged (38-42 weeks old) female mice were employed. Reproductively aged mice were divided into two groups, with one receiving the antioxidant, apocynin, 5 mM, in drinking water 7 weeks prior to sacrifice. Water bottles were protected from light and changed twice a week. At the selected ages, female mice were anesthetized with isoflurane and euthanized by cervical dislocation, and the ovaries were excised. One ovary was immediately frozen in liquid nitrogen and subsequently kept at -80°C for molecular studies and the other was fixed overnight, in 4% paraformaldehyde, for structural studies.

### 2.2. Tissue Processing for Histological Techniques

Fixed ovaries were dehydrated with the aid of increasing concentrations of ethanol and diaphanized using benzol. Impregnation and inclusion were carried out in paraffin, and 5 *μ*m thick sequential sections were mounted on poly-L-lysine-coated slides and dried overnight at 37°C. They were stored in plastic boxes to be used for all histological applications throughout this study.

### 2.3. Morphological Analysis of Ovarian Tissues

Ovarian sections for morphological examination were stained with hematoxylin & eosin (H&E) according to the following protocol. Slides were dewaxed twice with xylol and hydrated with decreasing concentrations of ethanol and water. Subsequently, slides were stained with Harris hematoxylin for 2 minutes and then stained with alcoholic eosin for 5 minutes. Finally, tissues were dehydrated with increasing concentrations of ethanol followed by two xylol passages. Slides were mounted in Entellan and air dried. Ovarian morphology was observed under light microscope equipped with a digital camera, and representative images at ovarian midsection were captured. Other ovarian sections were stained with Sudan Black 0.1% for lipofuscin examination. Slides were dewaxed and hydrated as previously described, stained with Sudan Black 0.1% for 20 minutes, dehydrated, and mounted.

### 2.4. Follicle Counting at Ovarian Midsection

H&E slides were used for follicle counting. The follicles were branded as primordial, primary, secondary, or antral, and *corpus luteum*. Follicles were classified as primordial or primary when oocytes were surrounded, respectively, by a single layer of squamous or cuboidal granulosa cells. Secondary follicles were identified by having more than one layer of granulosa cells with no visible antrum. Antral follicles are the ones that displayed small areas of follicular fluid (antrum) or a single large antral space. *Corpus luteum* were identified as intraovarian bund structures with morphologically homogeneous round cells, showing enhanced cytoplasm/nucleus ratio, when compared with granulosa cells, and deprived of oocyte. The number of follicles at ovarian midsection was obtained by calculating the mean counts of three ovarian midsections, representative of each animal, and a minimum of four animals per group was used.

### 2.5. Autofluorescence Lipofuscin Detection

Ovarian sections were dewaxed and hydrated as previously described. After washing in PBS, they were mounted in phosphate-buffered glycerol and observed under a fluorescence microscope (Carl Zeiss AxioImager Z1) equipped with a digital camera.

### 2.6. Fluorescent Immunohistochemistry

A fluorescent immunohistochemical technique was performed to detect protein carbonylation and nitration (markers of oxidative stress). Ovarian sections were dewaxed and hydrated as previously described. Slides were washed with phosphate-buffered saline (PBS) solution and permeabilized with 0.5% Triton™ X-100 in PBS for 5 minutes, followed by washing with PBS. For protein carbonylation assessment, an additional derivatization process was required before nonspecific signal blocking. The derivatization protocol was performed as previously published [25]. In brief, sections were incubated in 10 mM 2,4-dinitrophenylhydrazine (DNPH; Sigma-Aldrich) in 10% TFA for 10 minutes, the reaction stopped with 2 M Tris base pH 10, and slides were washed with PBS. Background staining was blocked with 2% bovine serum albumin (BSA) in PBS with 0.1% Tween 20 (PBS-T) for 1 hour at room temperature. Slides were then incubated with a rabbit polyclonal antibody recognizing DNP (1 : 250; Sigma-Aldrich) or a mouse monoclonal antibody recognizing nitrotyrosine (1 : 250; Santa Cruz Biotechnology) in PBS-T, overnight at 4°C. The following day, slides were washed in PBS-T, incubated with Alexa Fluor 488-conjugated anti-rabbit IgG secondary antibody (1 : 750; Molecular Probes) or in Alexa Fluor 568-conjugated anti-mouse IgG secondary antibody (1 : 750; Molecular Probes) in PBS-T for 1 hour at room temperature. Then, they were washed again, counterstained with 4′,6-diamidino-2-phenyl-indole (DAPI), and mounted. Slides were examined, and images were recorded under a fluorescence microscope (Carl Zeiss AxioImager Z1) equipped with a digital camera. Signal extension was quantified using ImageJ software by identification of the threshold cut point, with blind intervention of three operators. A minimum of four animals per group was used, and a minimum of two representative sections of the midovary was examined.

### 2.7. Fibrosis Evaluation in Ovarian Tissue

Picrosirius red histochemical technique was performed for quantification of tissue fibrosis. Ovarian sections were dewaxed and hydrated as previously described. Then, slides were stained with sirius red solution for 90 minutes, rapidly passed through 0.5% acidified water, and subsequently dehydrated, with increasing concentrations of ethanol, followed by two xylol passages. Slides were mounted in Entellan for further visualization and analysis. This technique stains collagen in red and cytoplasm in yellow. Red staining (collagen) was quantified using the ImageJ software by identification of the threshold cut point, with blind intervention of three operators. A minimum of four animals per group was used, and a minimum of two sections of the midovary was examined.

### 2.8. Real-Time PCR

Ovarian RNA extraction and purification was performed with TripleXtractor direct RNA kit (GRiSP) following the manufacturer's instructions. Total RNA was quantified by measuring the absorbance at 260 nm in a NanoDrop (Thermo Fisher Scientific), and RNA purity was evaluated by the ratio of absorbance at 260 and 280 nm. RNA samples were reverse transcribed to cDNA with the NZY First-Strand cDNA Synthesis kit (NZYTech). Real-time PCR was carried out using PowerUp SYBR Green Master Mix (Thermo Fisher Scientific) and specific primers (Table 1) in a StepOnePlus™ Real-Time PCR System (Applied Biosystems). Primers were designed using the online available specific mRNA sequences and Primer3 program. The derived sequences were submitted for a BLAST search to ensure exclusive alignment to the desired target genes. Amplification reactions were performed, in duplicate, according to conditions stated in Table 1. To check specificity, a dissociation curve was derived at the end of each run. Controls lacking reverse transcriptase were included to ensure no genomic DNA contamination during preparations. Results were normalized to 18S expression.

### 2.9. MicroRNA Quantification

Total RNA extraction was performed using the commercial Recover ALL™ Total Nucleic Acid Isolation Kit (Ambion), according to the manufacturer's instructions. In brief, for each sample, four paraffin-embedded ovaries were sectioned at 20 *μ*m thickness. Each sample was dewaxed in 100% xylene, washed in 100% ethanol, and incubated with protease and digestion buffer for 2 hours at 50°C, followed by 15 minutes at 75°C. Nucleic acids were isolated and incubated with DNase mix for 30 min at room temperature. After a final purification, 7 *μ*L of total RNA was reverse transcribed to cDNA using the MystiCq microRNA cDNA Synthesis Mix (Sigma-Aldrich Co.). In the process of cDNA synthesis, miRNAs were subjected to polyadenylation by poly(A) polymerase that catalysed the transfer of adenosine deoxynucleotides to the 3′ end. ReadyScript Reverse Transcriptase and other necessary reagents for cDNA synthesis were subsequently added to convert the poly(A) tailed microRNAs into first-strand cDNA using an oligo-dT adapter primer. The unique sequence at the 5′ end of the adapter primer allows amplification of microRNA cDNAs in real-time qPCR reactions using the MystiCq microRNA qPCR Universal Primer. Real-time PCR was carried out as previous described. Controls lacking reverse transcriptase were included to ensure no genomic DNA contamination during preparations, along with controls lacking poly(A) polymerase. Results were normalized to RNU1A (Qiagen) expression. Specific primers used were miR-21∗_1; miR-29c_1; miR-215_1; and miR-212-3p_1 miScript Primer Assay (Qiagen), with annealing temperature of 55°C.

### 2.10. Statistical Analysis

Arithmetic means are given with standard error of the mean (SEM). Statistical analyses were performed with GraphPad Prism 6.01 using one-way analysis of variance (ANOVA), followed by the Tukey posttest. A *P* < 0.05 was considered statistically significant.

## 3. Results

### 3.1. Age-Related Alterations in Ovarian Morphology

**Figure 1:**
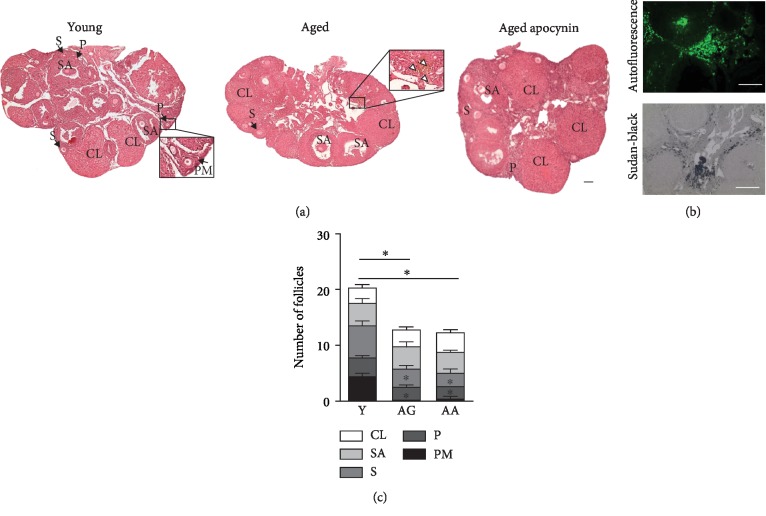
Ovarian structure and follicle number. (a) H&E-stained representative midovarian sections from young and aged mice. Note yellow-brown palely stained multinucleated cells, located in stroma of reproductively aged mice (white arrowheads). (b) Representative images of Sudan Black staining and autofluorescence in the ovaries of aged mice. Multinucleated cells stain intensely with Sudan Black and emit strong autofluorescence, indicating age-related deposition of oxidized proteins and lipids. Average quantification of autofluorescence area (*n* = 4‐5 per group). (c) Follicle distribution at midovarian sections of young and reproductively aged mice (*n* = 4 per group), showing a significant decrease in primary and primordial follicle number of older animals. Y: young; AG: aged; AA: aged apocynin; PM: primordial follicles; P: primary follicles; S: secondary follicles; SA: secondary antral follicles; CL: corpus luteum. Bars = 100 *μ*m. Data are presented as mean ± SEM. ^∗^*P* < 0.05, compared with young female mice.

To observe ovarian morphology, H&E-stained midorgan sections (Figure 1(a)) revealed follicles in various stages of development, occupying most areas of the ovary, but predominantly in the periphery. Central areas were occupied by a heterogeneous stroma that included vessels, bundles of extracellular connective tissue, and respective cells.

A unique population of stroma cells was detected only in the ovaries of reproductively aged mice. They were large and multinucleated, with pale cytoplasm containing a yellow-brown pigment (Figure 1(a)), which displayed autofluorescence (Figure 1(b)). Fluorescence quenching by staining with Sudan Black indicated that these were deposits of lipofuscin (oxidized lipids and proteins) (Figure 1(b)). Ovarian stromal cells with the abovementioned characteristics were consistently observed in reproductively aged mice and correspond to multinucleated giant macrophages [13, 26]. Follicle number was evaluated and, as expected, in reproductively aged mice, a significant reduction was noticed. This was due to a decrease in the number of primordial and primary follicles. Apocynin had no effect (Figure 1(c)).

### 3.2. Age and Antioxidant Supplementation Effect on Ovarian Oxidative Stress and Fibrosis

**Figure 2:**
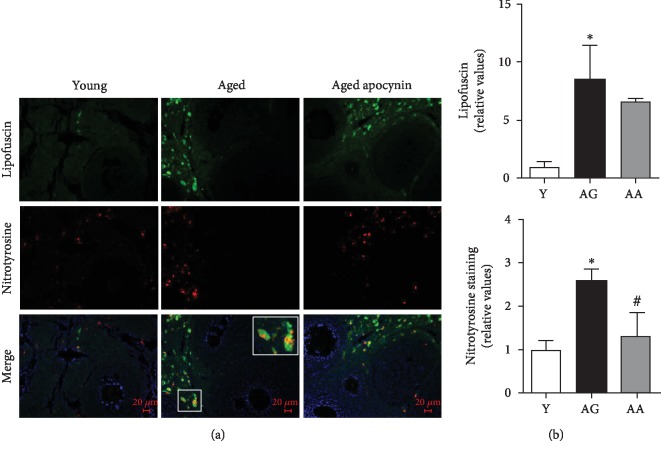
Protein nitration in the mouse ovaries evidenced by fluorescence immunohistochemistry, using a specific antibody. (a) Representative images of young, reproductively aged and apocynin-treated aged mice. Note tyrosine nitration staining and lipofuscin colocalization in the tissue. (b) Average quantification of lipofuscin and nitrated proteins stained area assuming young mice equals 1 (*n* = 4‐5 per group). Y: young; AG: aged; AA: aged apocynin. Data are presented as mean ± SEM. Data are presented as mean ± SEM. ^∗^*P* < 0.05, compared with young female mice; ^#^*P* < 0.05, compared with reproductively aged female mice.

**Figure 3:**
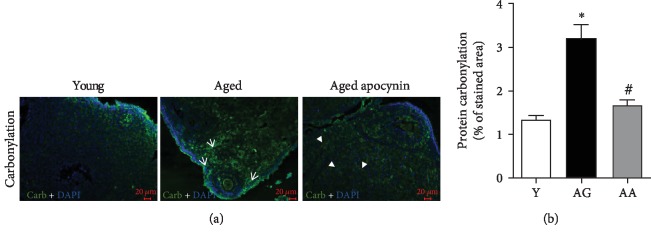
Protein carbonylation in the mouse ovaries evidenced by fluorescence immunohistochemistry, using a specific antibody for DNP. (a) Representative images of young, reproductively aged and apocynin-treated aged mice. Note the substantial carbonyl labeling increase in ovarian stroma cells (white arrows) and antioxidant-mediated amelioration (arrowheads). (b) Average quantification of stained area (*n* = 4‐5 per group). Y: young; AG: aged; AA: aged apocynin. Data are presented as mean ± SEM. ^∗^*P* < 0.05, compared with young female mice; ^#^*P* < 0.05, compared with reproductively aged female mice.

Tyrosine nitration and protein carbonylation were used to characterize ovarian oxidative stress (Figures 2 and 3). As shown in Figure 2, protein tyrosine nitration staining was found on the medullary stromal cells and partially colocalized with lipofuscin deposits. DNP immunoreactivity was observed in stromal cells, theca cells, oocytes, and corpus luteum (Figure 3). Reproductively aged females had increased expression of lipofuscin deposits (1.00 ± 0.41 vs. 8.53 ± 2.95), carbonylated (1.34 ± 0.09 vs. 3.17 ± 0.35), and nitrated proteins (1.00 ± 0.20 vs. 2.60 ± 0.26) (Figures 2(b) and 3(b)). The use of apocynin, that inhibits NOX-mediated superoxide production, resulted in a significant reduction in both carbonylated and nitrated proteins, to levels similar to those observed in the younger group (Figures 2 and 3).

**Figure 4:**
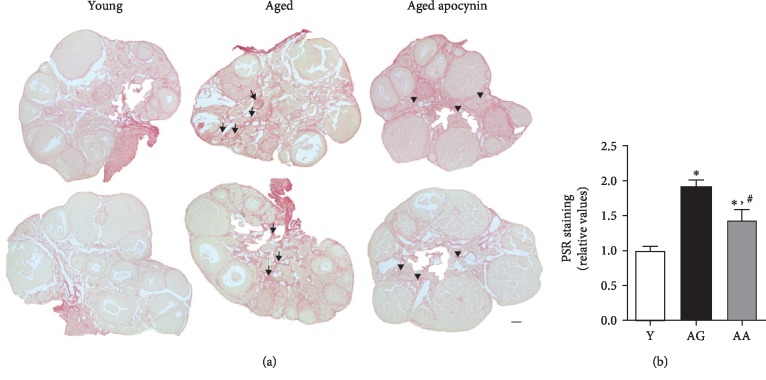
Fibrosis in mouse ovarian midsections stained by picrosirius red. (a) Representative images of young, reproductively aged and apocynin-treated aged mice. Note increased deposition of collagen fibers in the stroma of reproductively aged mice (arrows) and antioxidant-mediated amelioration (arrowheads). (b) Relative quantification of stained area assuming young mice equals 1 (*n* = 4 per group). Y: young; AG: aged; AA: aged apocynin. Bar = 100 *μ*m. Data are presented as mean ± SEM. ^∗^*P* < 0.05, compared with young female mice; ^#^*P* < 0.05, compared with reproductively aged female mice.

Oxidative stress appears to play a crucial role in the ethology of tissue fibrosis. Next, picrosirius red (PSR) histochemical technique was used to evaluate ovary collagen fibril deposition. Slight PSR staining was observed around follicles, blood vessels, and epithelium of the ovarian surface (Figure 4(a)). However, a network-like, intense PSR staining, characteristic of fibrotic foci, was only seen on the ovarian stroma and increased significantly with age (1.00 ± 0.12 vs. 1.92 ± 0.09) (Figure 4). In contrast, reproductively aged females treated with apocynin exhibited significantly reduced PSR staining (1.43 ± 0.15) (Figure 4(b)).

### 3.3. Age and Treatment Effect on Inflammation Factors and Collagen Expression

**Figure 5:**
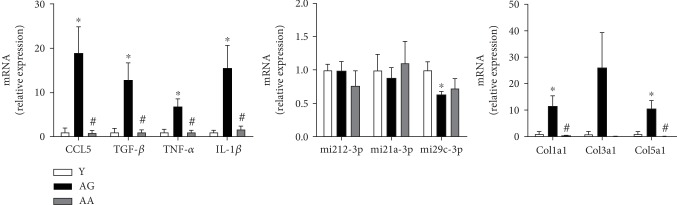
Expression of inflammation/fibrotic factors and extracellular matrix proteins. Relative mRNA and miRNA expression was calculated using the 2^*Δ*CT^ (ΔCT = CT_reference RNA_ − CT_target_), and values were expressed as fold change over young group (*n* = 4‐7 per group). Reproductive ageing is associated with an inflammatory and profibrotic ovarian microenvironment, increased levels of cytokines and collagens, and decreased level of the antifibrotic mi29c-3p RNA. Apocynin treatment reverts the expression of inflammation and fibrosis factors. Y: young; AG: aged; AA: aged apocynin. Data are presented as mean ± SEM. ^∗^*P* < 0.05, compared with young female mice; ^#^*P* < 0.05, compared with reproductively aged female mice.

**Figure 6:**
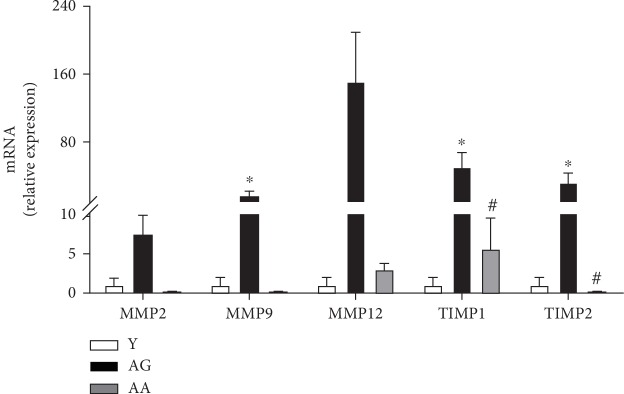
Expression of metalloproteinases and their tissue inhibitors. Relative mRNA expression was calculated using the 2^*Δ*CT^ (ΔCT = CT_reference RNA_ − CT_target_), and values were expressed as fold change over young group (*n* = 4‐7 per group). Reproductive ageing is associated with increased metalloproteinases expression and their tissue inhibitors. Apocynin treatment normalizes RNA expression. Y: young; AG: aged; AA: aged apocynin. Data are presented as mean ± SEM. ^∗^*P* < 0.05, compared with young female mice; ^#^*P* < 0.05, compared with reproductively aged female mice.

As previously mentioned, synthesis and secretion of various growth factors and cytokines are interlinked with OS in feedforward and feedback cycle mechanisms that might contribute to fibrosis via enhanced inflammation. Reproductive ageing was accompanied by a significant increase in several cytokines [(CCL5 (1.00 ± 0.92 vs. 18.93 ± 5.96), TNF-*α* (1.00 ± 0.66 vs. 6.82 ± 1.73), IL-1*β* (1.00 ± 0.40 vs. 15.55 ± 5.13)], including TGF-*β*1 (1.00 ± 0.90 vs. 12.85 ± 3.81) that is considered the most important cytokine modulating fibrosis signalling (Figure 5). All those molecules also regulate specific miRNA expression involved in fibrosis. Interestingly, as shown in Figure 6, miRNA29c-3p, a “master fibromiRNA,” was found to be downregulated in the ovaries of reproductively aged females (1.00 ± 0.13 vs. 0.57 ± 0.06), unlike miRNA212-3p (1.00 ± 0.11 vs. 0.86 ± 0.13) and 21a-3p (1.00 ± 0.25 vs. 0.76 ± 0.14) that were not affected by ageing. Expression of miRNA 29c-3p was inversely correlated with collagen expression. Both collagen types Col1a1 (1.00 ± 0.88 vs. 11.48 ± 3.88) and Col5a1 (1.00 ± 0.91 vs. 10.51 ± 3.10) were significantly increased in aged females. Apocynin treatment normalized cytokine and collagen expression to levels similar to those observed in younger females (Figure 5).

### 3.4. MMP/TIMP Expression

MMPs regulate not only ECM degradation but also bioavailability and activity of cytokines, chemokines, and growth factors. MMP activity is, in turn, regulated by its specific TIMPs. During reproductive ageing expression of MMP9, TIMP1 and TIMP2 were significantly increased [(1.00 ± 0.95 vs. 16.80 ± 5.63), (1.00 ± 0.97 vs. 51.62 ± 16.61), and (1.00 ± 0.96 vs. 33.09 ± 10.88), respectively] (Figure 6). Once more, apocynin dietary supplementation reversed age-related observed changes (Figure 6).

## 4. Discussion

The present study revealed that, during reproductive ageing, the ovary undergoes morphological and molecular changes related with a disturbance of the redox homeostasis, shown by increased lipofuscin content, protein carbonylation and nitration, and collagen deposition. Along with those changes, there was an age-related increase of inflammation and fibrosis, evidenced by higher relative expression of inflammation markers, MMPs, TIMPs, and a specific miRNA. Antioxidant supplementation with apocynin was capable of ameliorating features of the ovarian ageing process.

Over time, cells and tissues display structural changes that reflect age-related modifications in biomolecules and signalling pathways, eventually leading to tissue dysfunction [27]. Beyond time-related structural changes, the ovary is distinctly affected by additional cyclic ones, tightly controlled by hormonal variations. The mild, albeit continued, inflammatory nature of such alterations subjects the ovary to recurrent OS, inflammatory cell invasion, and fibrosis, especially intensified during ageing, when a decrease in antioxidant defences deepens the cellular redox imbalance [6–8, 28, 29]. The regulatory mechanisms contributing to age-related ovarian changes are still poorly known. Previously, we demonstrated that dietary supplementation with apocynin enhanced pregnancy outcomes at a latter reproductive age by improving the observed age-related decrease in mouse litter size and restoring decidua layer thickness [24]. In this work, apocynin was used to study its antioxidant properties in mitigating ovarian ageing effects.

Two features that distinguished young and reproductively aged ovaries were a unique population of multinucleated giant cells containing lipofuscin deposits and the number of follicles. In aged mice, a significant reduction in the number of primordial and primary follicles was observed, which agrees with the well-known time-related decline of the follicle resting pool [2, 30]. At this age, as expected, dietary supplementation with apocynin did not prevent the decline, because natural oocyte attrition had occurred. However, female fertility is not only dependent on the follicle pool but also on follicle and oocyte quality [31, 32], itself influenced by the ovarian oxidative microenvironment [13]. The ovarian stromal giant multinucleated cells present in reproductively aged mice were previously shown to be positive for a cell surface membrane protein—F4/80—highly expressed in macrophages [13, 20, 22]. Such observation supports the perception that they result from macrophage fusion. In the ovaries, macrophages are important contributors to the regulation of folliculogenesis and corpora lutea establishment and regression [33]. The unique presence of giant multinucleated cells in aged ovaries indicates that they associate closely to long-term effects on the tissue. Macrophage fusion is a hallmark of a low-grade chronic inflammatory condition, a recurrent oxidative challenge that potentiates phagocytic function for the disposal of cell debris [34, 35]. Such phagocytic cells are a source of factors that promote OS. Besides lipofuscin, itself a deposit of oxidized protein and lipid, our results suggest they also accumulate nitrated proteins. Probably, due to lipofuscin undegradable nature and inability to be removed from cells, apocynin treatment did not reduce its deposition, unlike protein carbonylation and nitration content.

The low-grade chronic inflammatory condition favours overproliferation of fibroblasts, excessive ECM deposition, and fibrosis [36]. In accordance with a previous study using two different strains of mice [13], the current study displays a significant age-related increase of fibrosis around follicles, blood vessels, and in the ovarian stroma. The observation that treatment with apocynin results in less ovarian fibrosis highlights the role of oxidative imbalance in its formation and suggests that NOX activity mediates local ROS production and susceptibility to fibrosis. NOX-derived ROS has, in fact, been associated with fibrosis in other organs such as the kidney [37], pancreas [38], and liver [39–41]. Moreover, as the inhibition prevents the enzyme cytoplasmic subunits from binding transmembrane complexes necessary for NOX1 and NOX2 activation (but not NOX4), it supports the view that the beneficial effects observed in the current study were mediated by inhibition of those two isoforms. Future works with specific NOX4 inhibitors will be useful to investigate its role in ovarian fibrosis.

Inflammation has an important role in fibrosis establishment by promoting a set of interactions between profibrotic and antifibrotic cytokines [42]. In the present study, increased expression of genes involved in immune cell response and recruitment was noticed. In this setting, macrophages are the main innate immune cells that release cytokines and chemokines to the local environment, of which, most impressively, NOX-mediated ROS production is an important modulator [43–45]. We observed a significant age-related increase in CCL5, an important chemokine in macrophage recruitment; IL-1*β* and TNF-*α*, proinflammatory cytokines involved in regulating endothelial cells in ECM production; and TGF-*β*1, a multifunctional cytokine highly responsible for ECM homeostasis through several pathways. Equally to fibrosis, apocynin reduced their expression to young mouse levels demonstrating, once more, that ROS play a master role in fibrosis and inflammation associated with reproductive ageing.

TGF-*β*1 involvement in ECM homeostasis includes the modulation of miRNAs that affect collagen deposition [46, 47]. Different types of miRNA with regulatory effects were recognized in mouse ovaries [48, 49]. In our study, the profibrotic miRNAs 21a-3p and 212-3p showed no differences with age. However, antifibrotic mi29c-3p, considered a “master fibrosis” regulator in several organs [47, 50–52], exhibited a significant reduction in the ovaries of reproductively aged females, which correlated with the significant increase in gene expression of Col1a1 and Col5a1. Surprisingly, apocynin attenuated, but not reverted, mi29c-3p decrease. Further studies are needed for a better comprehension of the mechanisms involved.

In addition, age-associated changes in MMPs and TIMPs can further contribute to fibrosis, as they modulate the activation of macrophages and fibroblast originated proinflammatory cytokines, as TNF-*α* and TGF-*β*1 [35]. Their roles in ECM remodelling as well as fibrosis establishment and progression are tissue specific. In reproductively aged ovaries, MMP9, TIMP1, and TIMP2 had a significant increase in gene expression. MMP9 is a gelatinase responsible for collagen degradation, including type I and V [53], that has been suggested to be directly regulated by TGF-*β*1 [54] and to take part in its activation [55] by cleaving its latent form [56]. TIMP1 and TIMP2 have profibrotic roles by inhibiting MMP activity and contributing to ECM accumulation [16]. However, the efficiency of MMP inhibition differs with each TIMP. This could explain the observed significant increase of MMP9, despite the observed aged-related significant increase in TIMP, further emphasizing the important role and relationship with TGF-*β*1. Our findings suggest that during reproductive ageing, the continued ROS-mediated inflammation disrupts ECM homeostatic processes inevitably ending in tissue fibrosis.

Overall, supplementation with apocynin ameliorated molecular and histological consequences of the ovarian ageing process. By diminishing ROS production, apocynin decreased tissue fibrosis, inflammation, and ECM deposition. This comes as a novel finding for the use of this antioxidant as, to our knowledge, no other work evaluated the capability of this compound in slowing down age-related ovarian reproductive ageing.

In summary, the present study provides evidence that structural features of ovarian ageing are consequence of local continued OS effects, with important negative impact on the ovarian stroma. Disruption of microenvironment, caused by a feedforward loop in which OS, inflammation, and ECM turnover dysregulation are important players, is believed to affect ovarian function and, ultimately, female fertility. Moreover, specific antioxidant supplementation can be seen as an important therapeutic way to ameliorate causes and effects of the ageing process induced by higher ROS production. Further investigation in this field, addressing apocynin effects on oocyte quality, may have major potential value to uncover fundamental biological mechanisms and devise therapeutic strategies.

## Figures and Tables

**Table 1:** List of primers used in RT-PCR reactions.

Primers		Sequence	Annealing temperature	Fragment (bp)
Col1*α*1	Fwd	5′-GACGCATGGCCAAGAAGACA-3′	60°C	85
	Rev	5′-CTCGGGTTTCCACGTCTCAC-3′		

Col3*α*1	Fwd	5′-AGCTTTGTGCAAAGTGGAACC-3′	58°C	114
	Rev	5′-ATAGGACTGACCAAGGTGGC-3′		

Col5*α*1	Fwd	5′-CCTGGTTCAGTGAATTCAAGCG-3′	60°C	81
	Rev	5′-TCATTTGTACCACGCCCACG-3′		

TGF-*β*1	Fwd	5′-ATTCCTGGCGTTACCTTGG-3′	60°C	120
	Rev	5′-AGCCCTGTATTCCGTCTCCT-3′		

TNF-*α*	Fwd	5′-CCCTCACACTCAGATCATCTTCT-3′	60°C	61
	Rev	5′-GCTACGACGTGGGCTACAG-3′		

IL-1*β*	Fwd	5′-TGACGGACCCCAAAAGATGA-3′	60°C	87
	Rev	5′-TGCTGCGAGATTTGAAGCTG-3′		

CCL5	Fwd	5′-TGCCCACGTCAAGGAGTATT-3′	60°C	84
	Rev	5′-ACTTGGCGGTTCCTTCGAG-3′		

MMP2	Fwd	5′-TGTCGCCCCTAAAACAGACA-3′	58°C	65
	Rev	5′-TGGGGCAGCCATAGAAAGTG-3′		

MMP9	Fwd	5′-CCTGGAACTCACACGACATCT-3′	62°C	72
	Rev	5′-CACGCCAGAAGAATTTGCCAT-3′		

MMP12	Fwd	5′-GGGCTGCTCCCATGAATGAC-3′	56°C	85
	Rev	5′-GTCATTGGAATTCTGTCCTTTCCA-3′		

TIMP1	Fwd	5′-GGTGTGCACAGTGTTTCCCTGTTT-3′	60°C	72
	Rev	5′-TCCGTCCACAAACAGTGAGTGTCA-3′		

TIMP2	Fwd	5′-GGATTCAGTATGAGATCAAGC-3′	55°C	145
	Rev	5′-GCCTTTCCTGCAATTAGATAC-3′		

18S	Fwd	5′-CGCCGCTAGAGGTGAAATTC-3′	60°C	67
	Rev	5′-CATTCTTGGCAAATGCTTTCG-3′		

Fwd: forward; Rev: reverse; bp: base pairs.

## Data Availability

The data used to support the findings of this study are available from the corresponding author upon request.

## Supplementary Materials

Supplementary Materials Supplementary Figure 1: low amplification images of protein nitration and lipofuscin deposition in mouse ovaries. Supplementary Figure 2: low amplification images of protein carbonylation in mouse ovaries.
