# Supplementary material for: Apocynin Dietary Supplementation Delays Mouse Ovarian Ageing
Source: Oxid Med Cell Longev. 2019 Oct 20;2019:5316984. doi: 10.1155/2019/5316984 (PMC6854951; doi:10.1155/2019/5316984)

Supplementary Figure 1 – Low amplification images of protein nitration and liposfuscin deposition in mice ovaries.

Supplementary figure 2 – Low amplification images of protein carbonylation in mice ovaries.

Supplementary Figure 1

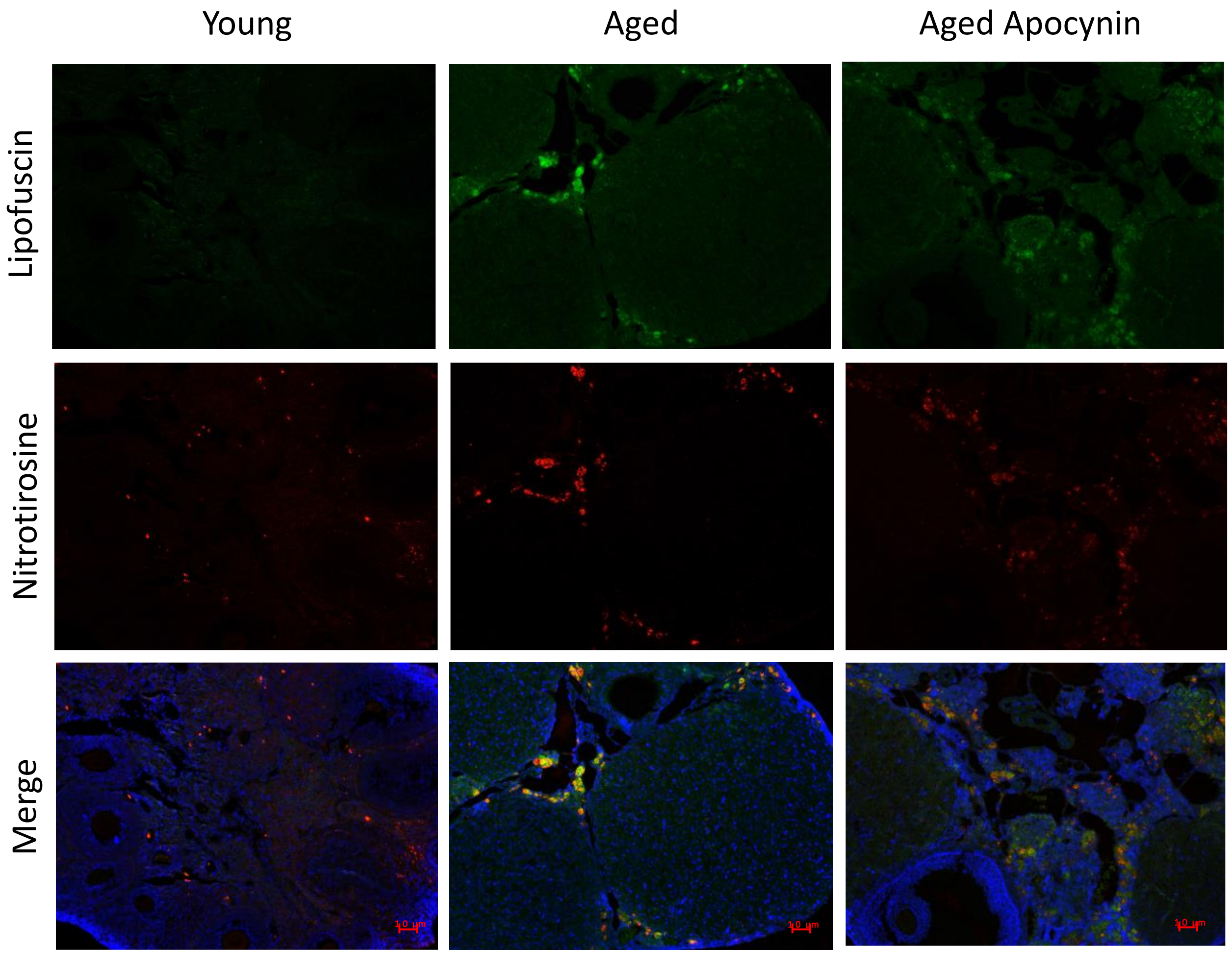

Supplementary Figure 2

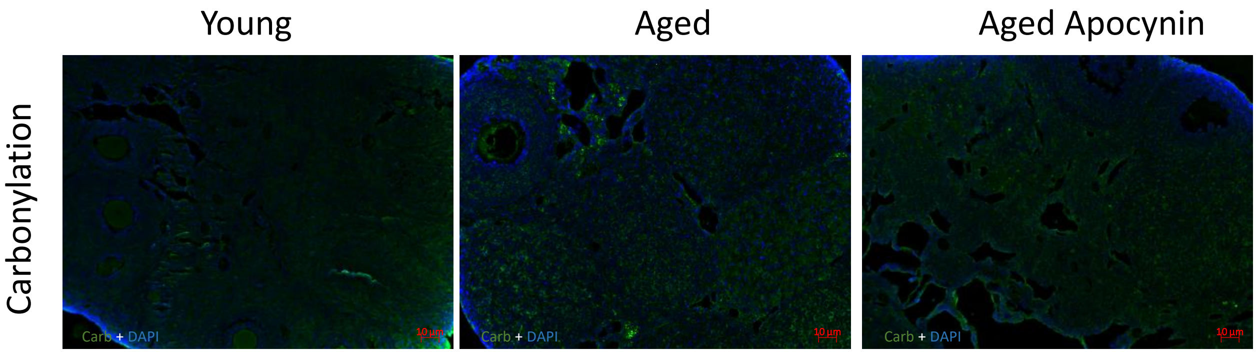

Supplement: Supplementary Materials — Supplementary Figure 1: low amplification images of protein nitration and lipofuscin deposition in mouse ovaries. Supplementary Figure 2: low amplification images of protein carbonylation in mouse ovaries. [file 5316984.f1.pdf]
